# 

**Published:** 1916-01

**Authors:** 


### SUBJECT INDEX TO VOLUME LXX

EVENT AND COMMENT.

American Institute of Dental Teachers, 102.

A New Method of Showing Clinics to Large Conventions. 201.

Annual Report of Forsythe Dental Infirmary, 203.

Another Gift to the Eastman Dental Infirmary, 51.

Chicago Dental Society, 101.

Dentistry in the European War, 552.

Focal Infection and Systemic Disease, 451.

Forsythe Loving Cup, 51.

Importing Programme Talent, 6.

Legalized Dental Hygienists, 251.

Memorial to Dr. H. A. Smith, 303.

Miller Memorial, 2.

National Research Laboratory, 4.

National Dental Association, Research Institute, 104.

New Dental School in New York City, 151.

Officers of the Ohio State Dental Society, 5.

Ohio State Dental Meeting, 1.

Passing of the Pioneers, 501.

Professional Instruction in Dentistry, 401.

Social Ideals, 301.

Surprising Dental Financing, 551.

The Eastman Idea, 103.

To Advertise or Not to Advertise, 52.

Unjust Discrimination Against the Army Dental Corps, 153.

United States Navy Dental Corps, 50[illegible].

What's in a Name, 53.

Who Should Teach Oral Hygiene, 4.

FORMAL PAPERS.

A Dental Efficiency Test. By Edward S. Barber, D.D.S., 320.

Agreement to Sell a Practice. By A. L. H. Street, 441.

Application and Dosage of Succinimid of Mercury. By. B. L. Wright, M.D., 85.

A New Method of Brushing the Teeth. By Henry Barnes, D.D.S., 304.

Are There Any Fixed Laws that Govern the Behavior of Amalgam? By N. K. Garhart, 62.

A Physician's Estimate of Modern Dentistry. By Woods Hutchinson, M.D., 27.

A Short Cut Method of Making Cast Gold Inlays. By Louis Herbst, D.D.S., 109.

A System of Making Porcelain Jacket Crowns. By L. E. Custer, D.D.S., 7.

Can I be a Better Dentist? .By C. F. Ash, D.D.S., 175.

Chronic Oral Infections and their Relations to other Diseases. By Thomas L. Gilmer, D.D.S., 154.

Commercialism in Dentistry, 440.

Conductive Anesthesia. By J. E. Nyman, D.D.S., 204.

Co-operation between Dentists and Orthodontists. By Martin Drury, D.D.S., 71.

Dental Infection and Systemic Disease, Treatment and Results. By E. E. Irons, M.D., 472.

Dental Laws and the State Boards. By Martin Dewey, D.D.S., 332.

Dental Hygienists and Preventive Dentistry, 434.

Dentists in the Army and Navy, 183.

Diagnostic Methods for Anesthesia. By F. A. Faught, M.D., 67.

Duty of Dentists in Cases where Choice Lies Between Removable and Fixed Bridges and Partial Denture. By George H. Wilson, D.D.S., 169.

Efficiency in Tooth Brushing. By Carl O. Enstrong, 60.

Further Facts Regarding Succinimid of Mercury as a Cure for Pyorrhœa, 280.

A Half Century of Antiseptic Surgery, 127.

High Pressure Anesthesia. By W. T. Jackman, 125.

Indirect or Model Methods for Inlays and Crowns. By E. B. Spalding, D.D.S., 386.

Injection Treatment of Neuritis with Hot Saline Solution, 592.

Interproximal Space and Contact Point. By C. N. Johnson, D.D.S., 366.

Liquid Petrolatum and its Trade Names, 330.

Mercury in Pyorrhœa. By Barton L. Wright, M.D., 566.

Modeling Compound vs. Plaster Paris. By Dayton Dunbar Campbell, D.D.S., 555.

Modern Field of Dental Practice, 351.

Mouth Infections, 522.

Musings of a Simpleton. By Hims Elf, 328.

My Prayer. By C. N. Johnson, 489.

New Method of Constructing Full Dentures. By W. Clyde Doris, 224.

Nitrous Oxide the Softest Volatile Anesthetic, 390.

Permeability of Normal Mucous Membrane by Bacteria. By P. R. Stillman, D.D.S., 432.

Personal Experiences. By L. P. Haskell, D.D.S., 521.

Preventive Dentistry. By W. I. Carlsen, 274.

Preventive Dentistry, 438.

Prevention, the Technique of the Future. By C. H. Gerrish, D.D.S., 21.

Proper Dietary for Dentists, W. W. Eldridge, M.D., 516.

Prevalence of Chronic Mouth Infections and their Management. By F. B. Moorehead, D.D.S., 453.

Pyorrhœa Alveolaris. By J. P. Buckley, D.D.S., 404.

Relation of Amebiosis to Pyorrhœa Alveolaris. By Julio Endelman, D.D.S., 324.

Remarks on Rigg's Disease. By Jules Sarrazin, D.D.S., 323.

Shall We Discontinue Devitalization? By Walter S. Kyes, D.D.S., 282.

Sir Frederick W. Hewitt, 105.

Some Recent Tendencies in Practice. By Arthur G. Smith, D.D.S., 118.

Some Refractories Used in Dentistry. By G. S. Millberry, D.D.S., 75.

Sterilization of Dental Instruments. By H. E. Hasseltine, D.D.S., 253.

Stop, 487.

Synthetic Porcelain. By Perry C. Baird, D.D.S., 426.

The Dentist and the Cancer Problem. By R. L. Byrnes, M.D., 308.

The Evidence Speaks for Itself. By Julio Endelman, 316.

The Principles Involved in Focal Infections as Related to Systemic Diseases. By Frank Billings, M.D., 461.

The Shortage of Platinum, 55.

The Supplee Impression Method. By E. R. Kibler, D.D.S., 504.

The Therapeutic Efficiency of Oral Preparations. By Herman Prinz, D.D.S., 353.

The Treatment of Narrow Nasal Passages. By A. Lohman, M.D., 14.

The Treatment of Pyorrhœa by the General Practitioner. By O. S. Clappison, D.D.S., 377.

Treatment of Pyorrhœa. By J. H. J. Upham, M.D., 429.

The Tri-State Meeting at Kansas City. By C. N. Johnson, D.D.S., 436.

Trip to the Panama-Pacific Dental Congress. By W. Claude Adams, D.D.S., 16.

Tuberculosis in the Mouth. By Hamilton Stillson, M.D., 423.

The Value of Oral Hygiene Lectures to the Public and Our Responsibility. By Thadeus P. Hyatt, D.D.S., 417.

What Should be the Fee for a Denture? By W. J. Holroyd, D.D.S., 227.

BIBLIOGRAPHY.

Appleton's Medical Dictionary, 250.

Food Analysis, 98.

Gould and Pyle's Cyclopedia of Medicine and Surgery, 540.

Mouth Hygiene, 541.

Internal Anatomy of the Face, 300.

Notes on Dental Metallurgy, 249.

Oral Abscesses, 591.

Peesos Crown and Bridge Work, 539.

Profitable Practice, 449.

State Board Questions and Answers, 249.

Who's Who in Dentistry, 591.

OBITUARY AND MEMORIALS.

G. V. Black, 48.

David W. Chever, 589.

George L. Field, 542.

Jacob W. Green, 287.

Charles H. Harroun, 150.

L. P. Haskell, 548, 549.

J. Ward House, 247.

Charles R. E. Koch, 546.

Abraham T. Metcalf, 543.

Norman G. Roech, 588.

W. X. Sudduth, 49.

Frederick Ward Putnam, 99.

Gordon White, 588.

INDENTS.

A Better Gold Inlay, 197.

A Dental Automobile for the English Army, 582.

Advantage of the Card System, 491.

Advertising, 190.

Ages of Carelessness, 491.

A Handy Matrix for Large Restorations, 447.

Alkaloidal Medication in Relation to Anesthesia and Analgesia, 138.

Amalgam Fillings, 91.

Amend English Dental Act, 391.

An Experience with Mercuric Succinimid, 447.

Amalgam not Poisoning Dentists, 133.

Answers to State Board Questions, 42.

Apoplexy During the Administration of Somnoform, 494.

Arsenic in Proximal Cavities, 89.

A Quick and Accurate Method of Adapting Porcelain Crowns, 191.

Aseptic Canal Treatment, 245.

Bacterius and Mercury in Treating Pyorrhœa Alveolaris, 134.

Bad Teeth and Ill Health, 586.

Better Dentists, 495.

Burnishing Silicate Fillings with Glass Rods, 399.

Carefulness in Diagnosis, 294.

Cast Inlays, 194.

Cause of Breakage in Platinum Pins, 234.

Causes, Results and Physiologic Treatment of Mouth Breathing, 144.

Cavity Varnish, 580.

Celluloid Matrix, 291.

Clasps, 335.

Cleaning Glassware, 31.

Clearing Modeling Compound from Trays, 391.

Cleanliness in Synthetic Operations, 339.

Commercializing the Profession, 94.

Comprehensive Dentistry, 233.

Confection for Administering Medicines, 93.

Conservatism in Extracting Diseased Teeth, 132, 575.

Conserving Children's Health, 137.

Chronic Poisoning by Emetin, 237.

Crown and Bridges vs. Partial Dentures, 236.

Danger of Infection, 146.

Deaths of Physicians in 1915, 96.

Dental Disaster, 289.

Dental Diseases and Diet, 445.

Dental Economies, 296.

Dental Hygiene, 398.

Dental Hygienists, 288.

Dental Libraries, 243.

Dental Reflex Pains, 235.

Dentists Exempt from War Service, 391.

Dentists' Health, 35.

Dentist Honored in England, 391.

Devitalizing for Crowns, 342.

Devitalizing Deciduous Teeth, 293.

Diagnosis, 393.

Dietetics and Caries, 393.

Dosage in Pressure Anesthesia, 396.

Early Orthodontic Treatment, 572.

Emetin—a Warning, 192.

Emetin and Heart Failure, 446.

Emetin in Pyorrhœa, 573.

Encroaching on Live Dentine, 31.

Enlarging Constricted Canals in Upper Bicuspids, 335.

Ether as an Antidote for Cocain, 335.

Ethics, 131.

Even Eggs may be Infected, 495.

Facility in Removing Teeth from Vulcanite, 141.

Failues I Have Had, 578.

Flasking, 236.

Function of Saliva, 145.

Glycerine, 447.

Gold Plating in Dentistry, 579.

Grinding Natural Teeth Painlessly, 195.

Hard to Prove, 131.

Heater for Water and Spray Bottles, 445.

Health Diet, 238.

Hillyer's Catechism, 493.

Hint for Using Felt Cones in Polishing, 136.

Injudicious Use of Hydrogen Dioxide, 32.

Immunity from Susceptibility to Dental Caries, 338.

Iodin as a Douche for Teeth, 584.

Ionization for Sterilization of Root Canals, 492.

Interrelations of Gastric and Salivary Digestion, 188.

Interpretation of Radiographs, 189.

Lack of Dentists, 391.

Local Anesthesia, 40.

Making Dies with Heavy Undercuts, 288.

Making Injections for Conductive Anesthesia, 292.

Making Plaster Models, 38.

Malignant Tumors of the Jaws, 33.

Medical College Attendance, 236.

Mouth Breathers, 136.

Necrotic Pulps, 195.

Novocain Injections, 37.

Novocain made in England, 581.

New York Dental Clinics, 237.

One Method of Treating and Filling Root Canals, 492.

Oral Hygiene in the Chesterfield Letters, 340.

Perforation of Root-canal Walls, 583.

Plates Rather than Bridges, 587.

Preserve the Pulps, 33.

Prevent Rusting of Steel Needles, 135.

Protecting the Gum Margins in Partial Dentures, 294.

Pyorrhœa a Preventable Disease, 491.

Quits Practice, 136.

Radio-dermititis from Diagnosing Diseased Teeth, 397.

Repairing an Inlay Margin, 585.

Repairing Hole in Gold Crown, 132.

Rapid Disinfection of Hands, 193.

Reflex Irritations, 443.

Replacing a Bridge Facing, 32.

Relation of Dentistry to Medicine, 36.

Responsible for Work Done by Assistants, 445.

Resting, 232.

Restoring Occlusal Surfaces with Amalgam, 34.

Root-canal Filling, 89.

Root-canal Filling by Two Methods, 191.

Root-canal Work, 581.

Root Filling, 336.

Rugae on Plates, 191.

Russian Women Dentists, 143.

Sanitation in Schools, 90.

Sciatica, 394.

Sealing Medicines in Teeth, 194.

Separating Modeling Compound Impressions, 199.

Septic Wheel Brushes, 192.

Shortage of Dentists in Europe, 92.

Silicate Fillings, 31.

Silver Plating Solution, 147.

Sir William Osler on Wounds and Disease in War, 580.

Sloppy Amalgam, 342.

Softening Mouldine, 140.

Soldering Flux, 140.

Some Dental Uses for Rice Flour, 291.

Sterilizable Washers for Hypodermic Syringes, 192.

Stypticin Tablets, 89.

Succinimid of Mercury, 131, 137.

Succinimid of Mercury in Treatment of Pyorrhœa Alveolaris, 582.

Synthetic Crowns, 585.

Teething as a Cause of Symptoms in Infants, 240.

Testing Root-canals for Cleanliness, 193.

Tightening Old Plates, 141.

The Care of Children's Teeth, 573.

The First Plaster Impression, 296.

The Joy of Intelligence, 89.

The Pathologic Significance of Impacted and Unerupted Teeth, 392.

The Pathologic Oral Cavity, 571.

The Use of Floss Silk in the Dental Toilet, 293.

Think, 444.

To Avoid Bubbles in Casting, 392.

To Give a Facing a Light Gray or Blue Shade, 586.

Toleration, 141.

To Obtund Dentin, 584.

To Prevent Models from Breaking, 140.

To Relieve Pain in Incipient Abscess or Neuralgia, 448.

To Remove Model from Articulator, 572.

Treatment of Pyorrhœa, 198.

Tropo Cocain in Spinal Anesthesia, 235.

Use of Clasps, 294.

Varnish for Silicate Cement Fillings, 193.

Vulcanite and Metal Plates, 141.

Wax Model Additions, 292.

Why Not Live Longer? 244.

Why We Prematurely Break Down, 90.

BIOGRAPHIC.

Adams, J. F., 141.

Adams, W. C., 16.

Allen, C. C., 445.

Ash, C. F., 14, 175.

Baird, P. C., 426.

Baldwin, C. F., 91.

Barber, E. S., 320.

Barnes, Henry, 304.

Best, E. S., 335.

Biddle, J. F., 197.

Billings, Frank, 461, 484.

Black, G. V., 48.

Bledsoe, C. W., 583.

Bleiler, H., 132.

Blue, J. B., 192.

Bodecker, C. F., 339.

Boys, R. S., 193.

Brown, M. M., 192.

Buckley, J. P., 404.

Burgess, Dr., 34.

Byrnes, R. L., 308.

Campbell, D. D., 555.

Carleson, W. I., 274.

Chisolm, G., 199.

Cigrand, B. J., 296.

Clappison, O. L., 377.

Converse, A. E., 491.

Copelan, C. S., 140.

Cox, Geo. E., 448.

Cummer, M. E., 294, 492.

Custer, L. E., 7.

Davis, Clude, 224.

Dewey, M., 71, 332.

Dolman, W. H., 137.

Dunning, W. B., 298, 584.

Eldridge, W. W., 516.

Endelman, Julio, 190, 237, 316, 324

Engstad, J. E., 336.

Engstron, Carl O., 60.

Entriken, H. L., 191.

Evans, E. T., 291, 445.

Faught, F. A., 67.

Fautus, B., 94.

Fellman, W. O., 342.

Field, Geo. L., 542.

Fisher, W. C., 481.

Forsythe, C. H., 491.

Frank, R., 193.

Gabell, D., 294.

Garhart, N. K., 62.

Gerrish, C. H., 21.

Gibbin, F. E., 293.

Gilmer, Thomas L., 154.

Gildersleev, Dr., 32.

Grant, W. H. R., 397.

Green, J. W., 287.

Gregg, G. T., 38.

Haskell, L. P., 335, 521, 548.

Hasseltine, H. E., 253.

Hayes, G. B., 553.

Herb, I. C., 139.

Herber, Carl, 40.

Herbst, Louis, 109.

Herrick, C. A., 342.

Hims, Elf, 328.

Hinman, H., 296.

Holroyd, W. J., 227.

Hopkins, J. C., 89.

House, J. Ward, 247.

Hutchinson, Woods, 27.

Hyatt, T. P., 417.

Irons, E. E., 472.

Jackman, W. T., 125.

Johnson, C. N., 366, 437, 490, 549.

Johnston, S. E., 240.

Kaplan, A. H., 31.

Kelly, W. S., 141.

Kibler, E. R., 504.

Kirk, E. C., 31, 444.

Klein, J. A., 194.

Koch, Chas. R. E., 546.

Kusel, Dr., 131.

Kyes, W. S., 282.

Lampert, E. E., 584.

Linsley, J. C., 288.

Lohman, A., 14.

Lyons, C. J., 393.

McCollough, Dr., 35.

Manning, P. R., 493.

Mayo, C. H., 36, 495.

Maskee, G. M., 398.

Merritt, A. H., 492.

Metcalf, A. T., 543.

Millberry, G. S., 75.

Moorehead, F. B., 453, 486.

Newkirk, G., 292.

Nyman, J. E., 204, 482.

O'Connell, J. W., 340.

Ottolengui, R., 194, 337, 587.

Pollock, J., 492.

Price, W. A., 483.

Prime, J. M., 292.

Prinz, H., 353.

Quinby, M., 393.

Reinhart, R. J., 90, 92.

Riethmuller, R. H., 135.

Ruzicka, M., 392.

Sarrazin, J., 323.

Sexton, H. C., 44.

Sherman, W. G., 198.

Silverman, S. C., 42.

Skinner, E. H., 291.

Sloan, A. C., 89.

Smedley, V. C., 132.

Smith, A. G., 118.

Smith, B. H., 45.

Spalding, E. B., 386.

Spain, C. J., 235.

Stillman, P. R., 434.

Stillson, Dr., 423.

Street, A. L. H., 441.

Sudduth, W. X., 49.

Supplee, S. G., 143.

Taylor, L. C., 136.

Thornton, J. W., 342.

Upham, J. H. J., 429.

Werts, H. C., 192.

Wilkes, A. M., 191.

Wilson, G. H., 169.

Wilkinson, W. S., 447.

Wolf, B., 135.

Woolgar, C. H., 292.

Wright, B. L., 85, 566.

### ANNOUNCEMENTS.

American Institute of Dental Teachers, 46, 200, 538.

American Society of Orthodontists, 148, 299.

Buffalo University Alumni Association, 47.

Choyes Dental Club, 400.

Chicago Dental Society Clinics, 47.

Clinics of National Dental Association, 343.

Connecticut State Dental Society, 150, 200.

Exhibit of Dental Manufacturer's Club, 100.

Illinois State Board of Examiners, 500.

Illinois State Dental Society, 200.

Indiana State Dental Society, 200.

Information for U. S. Navy Candidates, 528.

Lake Charles, La., Loves Company, 148.

Louisiana State Dental Society, 299.

Michigan State Board of Examiners, 200, 499.

Michigan State Dental Society, 148, 246.

Missouri, Kansas and Oklahoma Tri-State Meeting, 45.

National Dental Association, 299, 497.

National Association of Dental Faculties, 45, 246.

National Association of Dental Examiners, 299.

Nebraska State Board of Examiners, 246.

New Jersey State Dental Society, 149.

New York State Dental Society, 149.

Northern Ohio Dental Association, 100, 150, 200, 246.

Ohio State Dental Society, 499, 538.

Preparedness League of American Dentists, 497.

Research Institute of N. D. A., 349.

St. Louis Dental Society, 399, 498, 536.

Tennessee State Board of Examiners, 246.

Washington University Alumni Association, 46.

West Virginia State Board of Dental Examiners, 299.

### Publishers' Special Announcements

Advertising creates for the article advertised a recognized place among the articles of its kind for its particular use and has the efficacy to influence not only users of the particular article, but also people who do not use and have no use for the article. Every one knows that Mr. Gillette makes a safety razor; even the ladies know that, and now that he makes one for the ladies, the gentlemen likewise already know it. Advertising does this. Every dentist knows that The Sam'l. A. Crocker Co. make a rolling mill, even if he does not want one, being located in a city near enough to a local dental depot to have access to one of their mills. Advertising conveys this knowledge. The article advertised obtains the recognition of the people. Recognition leads to sales and sales to repeat orders.

If in doubt, ask for the advertised brand and it will always be good.

---

There is but one month left now in which to obtain subscriptions for the Dental Register before the prize contest closes on January 31, 1916. We trust all dental salesmen will continue the good work and turn in their accumulated orders on time.

---

FOR SALE—Dental Practice and complete equipment in town of 40,000, in Southern Ohio. Disposing of office on account of illness. Write for particulars to The Sam'l A. Crocker Co., Box 838, Cincinnati, Ohio.

---

FOR SALE—Dental Office, Practice and Equipment. Established patronage. Address Estate Dr. F. W. Brammer, Ironton, Ohio.

---

Subscriptions to the Dental Register will be received at office of The Sam'l A. Crocker Co. Price per year $1.00. Dental Dealers throughout the United States receive subscriptions for The Dental Register. Send your orders for balance of 1915 and 1916 through your dealer or direct. Founded 1846, now in 69th year. The Dental Register is the most reliable and conservative Dental Magazine published. Send in your orders now.

### ANNOUNCEMENTS

NATIONAL ASSOCIATION OF DENTAL FACULTIES—IMPORTANT POSTPONEMENT. The meeting of the National Association of Dental Faculties which was to have been held in Minneapolis, January 28-29, 1916, has been postponed to meet in Louisville in July, 1916. The exact dates will be announced later.

B. HOLLY SMITH,
*Chairman Executive Committee.*

---

THE TRI-STATE POST GRADUATE DENTAL MEETING—MISSOURI, KANSAS AND OKLAHOMA. The State Dental Societies of Kansas, Oklahoma and Missouri wish to announce to the dental profession that they will conduct a joint post-graduate dental meeting in Kansas City, Mo., the week of March 20-26, 1916.

In making this announcement we believe it to be the most important step, from a dental educational standpoint, ever undertaken by any state dental societies. The meeting is to be conducted along the lines of the "Oklahoma Way" or the "Teaching System." At this writing several of the foremost teachers of the dental profession have been secured for lectures, among whom are: Dr. M. L. Rhein, of New York; Dr. Richard Riethmueller, of Philadelphia; Dr. C. N. Johnson, of Chicago; Dr. Weston A. Price, of Cleveland; Dr. Thos. P. Hinman, of Atlanta; Dr. Forrest H. Orton, of St. Paul; Dr. J. P. Buckley, of Chicago. Some noted medical man's name will be added to this list of lecturers.

Doctor Rhein will take up the subject of root canal work; Doctor Riethmueller, anesthesia and analgesia, both local and general; Doctor Johnson, some phases of operative dentistry; Doctor Price, some recent research work;

Doctor Hinman, plastics (amalgam and cements); Doctor Orton, some phases of crown and bridge work, and Doctor Buckley, materia medica, therapeutics and pyorrhea.

Each person attending this meeting will be required to pay a membership fee of five dollars, and only dentists in good standing in their state society will be eligible to take this post-graduate course.

The Tri-State Bulletin and a program will be mailed upon request to anyone desiring to keep posted on the details of this event. For any information, address the secretary of the organization committee.

CHARLES CHANNING ALLEN, *Chairman*,
10th St. and Troost Ave., Kansas City, Mo.
C. R. LAWRENCE, *Secy.-Treas.*, Enid, Okla.

---

WASHINGTON UNIVERSITY DENTAL ALUMNI ASSOCIATION. Come home to the "Golden Jubilee" of the Washington University Dental School, Feb. 21st and 22nd.

The meeting will be held at the Dental School, 29th and Locust Sts.

Prominent educators will present an excellent program and the manufacturers' exhibits will be unsurpassed.

---

AMERICAN INSTITUTE OF DENTAL TEACHERS. The annual meeting of the American Institute of Dental Teachers will be held at Hotel Radisson, Minneapolis, Minn., January 25, 26 and 27, 1916.

There will be a number of interesting papers, reports and discussions by prominent dental educators. All dental teachers are cordially invited to be present.

J. F. BIDDLE, *Secretary*.
517 Arch Street, N. S. Pittsburgh, Pa.

ANNUAL CLINIC CHICAGO DENTAL SOCIETY. JANUARY 28-29, 1916, HOTEL LA SALLE, CHICAGO. The annual clinic of the Chicago Dental Society bids fair to surpass in efficiency and enthusiasm our splendid clinics in the past. A notable list of clinicians has been selected and arrangements are under way for a clinic which will place before our guests and members the best in modern scientific dentistry. The evening meeting on Friday, January 28, will mark an epoch in dentistry. The banquet will be held on Saturday evening, January 29.

Reservations for rooms at the La Salle Hotel, Chicago, may be made any time. Banquet tickets may be had by addressing Dr. S. W. Fahrney, 25 E. Washington St., Chicago. The official program will be issued Jan. 1. Exhibitors desiring space may communicate with Dr. L. Strong, 30 N. Michigan Ave., Chicago.

FREDERICK B. MOOREHEAD, President,
PERCY B. D. IDLER, *Secretary,*
30 N. Michigan Ave., Chicago.

---

BUFFALO ALUMNI MEETING. The Annual Alumni Meeting of the Dental Department, University of Buffalo, will be held at the Iroquois Hotel on Friday and Saturday, February 18 and 19, 1916.

We shall have with us this year Dr. Elmer S. Best of Minneapolis, who will bring to you an interesting essay and clinic on "New Methods of Root Canal Treatment," also Dr. Alfred S. Walker of New York City, who will clinic both days of the meeting on the following subjects, viz:

(a) Ionic Medication in Dentistry;

(b) The Faradic Coil as an aid in determining the vitality of the Pulp.

In addition to the above named clinicians we have several more from a distance and a good array of local talent.

All of the exhibit space is taken so you can be assured that the meeting will be up to the standard in this respect.

Don't forget that on Friday evening we shall have our usual dinner and a good social time.

BUSINESS COMMITTEE—
G. E. Jackson,
J. L. Cleveland,
T. A. Hicks.

